# Lateral Flow Test System to Control Total Content of Muscle Tissues in Raw Meat Products

**DOI:** 10.3390/s22249724

**Published:** 2022-12-12

**Authors:** Elena A. Zvereva, Olga D. Hendrickson, Boris B. Dzantiev, Anatoly V. Zherdev

**Affiliations:** Bach Institute of Biochemistry, Research Center of Biotechnology of the Russian Academy of Sciences, Leninsky Prospect 33, 119071 Moscow, Russia

**Keywords:** lateral flow assay, skeletal troponin I, immunoglobulin Y, colloidal gold, food control

## Abstract

Assessment of the composition of meat-containing products is the task in demand due to their frequent deviations from declared recipes. The paper presents the developed test system for immunochromatographic determination of total meat content. The assay is based on the simultaneous use of monoclonal antibodies, which specifically interacts with mammalian skeletal troponin I, and polyclonal antibodies, which specifically detect bird immunoglobulin Y. To integrate the detection of both types of meat by the same test strip, the antibodies are mixed in the analytical zone of the test strip and in complex with a gold nanoparticle label. The chosen ratios of the antibodies for both mixtures provide the same contribution of different types of mammalian and bird raw materials of muscle tissues to the label binding. The test system demonstrates suitability for products containing beef, pork, rabbit, lamb, chicken, and turkey meat. The minimal detectable content of meat in samples is 0.1%. The samples for the testing are diluted 100 times, thus eliminating matrix effects, and providing high reproducibility of the color intensity for extracts of different compositions. The obtained results allow the recommendation of the developed test system for rapid on-site control of meat products.

## 1. Introduction

Meat is an important component of human diets as a source of proteins, fats, vitamins, microelements, etc., [1,2,3], but sometimes it causes negative consequences. Wide variety of biologically active components in meat makes it an important modulator of physiological processes. Meat consumption, taking into account personal characteristics, ensures the maintenance of an active lifestyle and the effectively working protective systems of the organism [4,5].

Falsified meat products may pose a danger to human health or violate religious requirements [6,7]. The interest of manufacturers to reduce the cost of production may lead to deviations from declared recipes. These violations include the replacement of expensive types of meat with cheaper ones [8] or the reduction of the declared meat content [9]. Therefore, tasks in the control of meat product composition combine the identification of used species [10] and the determination of total content for muscle tissues from different species. The guaranteed content of meat raw materials in purchased products provides consumers with valuable food sources in terms of biological activity due to the various physiological effects of muscle tissue components [11,12,13].

The composition of meat products is characterized by a wide range of analytical methods: histological analysis, electrophoresis, various spectroscopic and chromatographic techniques, polymerase chain reaction, etc., [10,14,15,16,17]. However, the information obtained with their help is often limited by data on the presence or absence of certain components, whereas quantitative assessment of the composition requires additional complex, time-consuming and labor-intensive actions [6]. Immunochemical methods of analysis, first of all, the most widespread enzyme immunoassay (EIA) and immunochromatographic analysis (ICA), have undeniable advantages such as low cost and simple preparation of samples. Moreover, immunochromatographic test strips can be used without additional equipment and so applied in out-of-laboratory conditions [18]. The basis for the successful implementation of immunoanalytical techniques is the choice of an antigenic molecular marker for specific recognition of target food compounds. Such markers may be specific to some tissue (muscles, connective tissue, blood) [19,20,21], some species, or some systematic group of organisms [19,20,21,22,23,24]. The described immunochromatographic test systems allow detection or evaluation of the content of a specific kind of meat source, for example, pork meat [25] or poultry meat [24], but not the total sum of different meat ingredients. The given task is non-trivial in terms of methodology, forasmuch as the same response should be achieved for different compositions with the equal sum of several meat compounds.

In our previous works, troponin I (TnI) [26] and immunoglobulin Y (IgY) [24] were characterized and successfully applied to identify animal and bird muscle tissues, respectively. Their efficiency as biomarkers for detection of muscle tissues is determined by their specific immune recognition by available antibodies and high content in comparison with other potential biomarkers [27,28] that provides intense signal in ICA. Thus, the aim of the study was to develop a test system using skeletal troponin I and immunoglobulin Y as detected biomarkers and integrate their impact in the assay results for the determination of the total content of various animal and bird sources in meat products. ICA was chosen for the development of the existing immunoanalytical techniques since it transfers the control procedure outside the laboratories and provides information about testing results rapidly.

## 2. Materials and Methods

### 2.1. Chemicals

Chloroauric acid, bovine serum albumin (BSA), sodium citrate, Tween-20, Triton X-100, sucrose, and sodium azide were obtained from Sigma Chemicals (St. Louis, MO, USA, sial.com, accessed on 3 November 2022). D-biotin-N-hydroxysuccinimide ester was obtained from ICN Biomedicals (Irvine, CA, USA). Porcine skeletal troponin I and monoclonal antibodies (MAb) against TnI clones 7G2, C5, and 6F9 were purchased from HyTest (Turku, Finland). Rabbit anti-mouse immunoglobulins (RAMI), goat anti-rabbit immunoglobulins (GARI), and rabbit anti-chicken immunoglobulins G (RACI) were obtained from Imtek (Moscow, Russia). Peroxidase-labeled antibodies against mouse immunoglobulins G were obtained from Jackson ImmunoResearch (Cambridgeshire, UK). One-component substrate solution of 3,3′,5,5′-tetramethylbenzidine (TMB) was from Immunotech (Moscow, Russia). All salts, solvents, and other chemicals were from Khimmed (Moscow, Russia). Purity of the used reactants accorded to at least reagent grade. ELISAs were conducted on Costar 9018 96-well polystyrene microplates (Corning, NY, USA).

### 2.2. Biotinylation of Antibodies

Monoclonal antibodies to TnI, clones 7G2, C5, and 6F9 were preliminarily transferred into 50 mM K-phosphate buffer, pH 7.4, with 0.1 M NaCl (PBS) by dialysis in centrifuge tubes containing cellulose acetate filters (Spin-X, Corning Costar, NY, USA) at 10,000× *g* for 15 min.

Covalent binding of biotin to the antibodies was performed at a ratio of 10:1 (mol/mol) in the accordance with [29]. Namely, a solution of biotin-N-hydroxysuccinimide ester in dimethyl sulfoxide (3.1 mg/mL) was added to antibody preparation and incubated at room temperature for 2 h. The obtained biotinylated antibodies were dialyzed against PBS three times using Amicon Ultra Centrifugal Filters 10 K (Merck Millipore, Burlington, MA, USA) via 15-min centrifugation at 10,000× *g*.

### 2.3. Sandwich ELISA

The assay was performed as described in [14]. The sample successively interacted with antibody 7G2 or C5 or 6F9 immobilized in microplate wells (2 μg/mL in PBS) and with biotinylated antibody 7G2 or C5 or 6F9 (2 μg/mL in PBS containing 0.05% Triton X-100 (PBST)) used for the detection. Then streptavidin–horseradish peroxidase conjugate diluted 1:5000 was introduced into the wells. All steps were carried out in PBST containing 0.5 M KCl and lasted 1 h. After completion of each step, the microplate was washed four times with PBST. To detect the formed immune complexes, 100 μL of substrate solution TMB was added to the wells, incubated for 15 min, and 1 M H_2_SO_4_ was added. The optical density of the peroxidase reaction product was measured at 450 nm by Zenyth 3100 reader (Anthos Labtec Instruments, Wals, Austria).

### 2.4. Preparation of Gold Nanoparticles and Their Characterization

Gold nanoparticles (GNPs) with an average diameter of 25 nm were synthesized by reducing chloroauric acid using sodium citrate, as described in [30]. A solution of chloroauric acid (1.0 mL, 10 mg/mL) was added to 97.5 mL of water, heated to boiling and sodium citrate (1.5 mL, 10 mg/mL) was added. The preparation was boiled for 30 min, cooled, and then stored at 4 °C. The preparation was characterized by transmission electron microscopy (TEM) using a CX-100 microscope (Jeol, Japan) as reported in [26].

### 2.5. Immobilization of the Antibodies on the GNPs

The antibody–GNP conjugates were prepared, as described in [24,26]. MAbs 7G2, 6F9, C5, and RACI were dialyzed against 10 mM Tris-HCl buffer, pH 9.0, and added to AuNPs (OD_520_ = 1, pH 9.0). For conjugation with the GNPs concentrations of monoclonal antibodies 7G2, 6F9, and C5 were 5 μg/mL, and concentration of polyclonal antibodies RACI was 10 μg/mL. A series of 7G2 + RACI, 6F9 + RACI, and C5 + RACI conjugates were synthesized, in which the weight/weight ratio of MAb to TnI, and RACI was 50/50, 20/80, and 80/20%. The mixtures were stirred at room temperature for 45 min, BSA solution (10%) was added to a final concentration of 0.25% and incubated for 15 min. Conjugates were separated by centrifugation at 13,400× *g* for 20 min at 4 °C and re-suspended to an OD_520_ = 15 in 10 mM Tris-HCl buffer, pH 8.5, containing 1% BSA, 1% sucrose, and 0.05% sodium azide.

### 2.6. Production of Test Strips

To prepare test strips, a CNPC-SS12 working membrane with a 15 μm pore size, a GFB-R4 sample membrane, and an AP045 adsorption membrane (all from Advanced Microdevices, Ambala Cantt, India) were used. Antibodies were applied on the membranes (0.1 µL per mm width) using an Iso-Flow dispenser (Imagene Technology, Hanover, NH, USA). All applications were made in PBS.

*Individual test for TnI.* The analytical zone was formed using antibodies 6F9 (2.5 mg/mL) and the control zone was formed using RAMI (0.5 mg/mL in PBS).

*Individual test for IgY.* The analytical zone was formed by RACI (1 mg/mL) and GARI (0.5 mg/mL) was applied in the control zone.

*Combined test.* The analytical zone was formed using a mixture of antibodies 7G2, C5, or 6F9 (1.0, 2.0, or 1.75 mg/mL) with RACI (0.5, or 1.0 mg/mL), and the control zone was formed using a mixture of RAMI (0.33 mg/mL) and GARI (0.33 mg/mL).

After dispensing, all membranes were dried for at least 20 h, fixed on plastic support, and then cut into strips (3.0 mm width) using an Automatic Cutter (KinBio, Shanghai, China). The obtained test strips were stored at room temperature.

### 2.7. Sample Preparation

Raw chicken, turkey, pork, beef, goat, and rabbit meat were purchased in supermarkets. To obtain mixtures of different compositions, minced chicken and mammalian meat were combined (25%/75%, 50%/50%, 75%/25%, 90%/10%, 95%/5% g/g). Moreover, meat/not-meat mixtures were prepared by addition of minced chicken and pork meat to non-meat compounds.

For extraction in the accordance with [24], 5 mL of extraction buffer (PBS containing 0.1% Triton X-100 and 0.5 M KCl) was added to 250 mg of homogenized meat sample. The mixture was intensively stirred for 15 min, sonicated in an ultrasonic bath for 10 min, and then centrifuged for 10 min at 5000× *g*. The supernatants were collected and stored at –18 °C.

### 2.8. Performance of the ICA

In the microplate wells, 100 µL of the sample and 0.5–2 µL of the corresponding antibodies-GNPs conjugate (OD_520_ = 15) were mixed and incubated for 3 min. Test strips were immersed into the solutions, incubated for 15 min, and scanned using the CanoScanLiDE 90 scanner (Canon, Japan) with a 600 dpi resolution. TotalLab (TotalLab, Newcastle upon Tyne, Gosforth, UK) software was used to process the resulting images and to estimate the color intensity of the formed lines.

### 2.9. Data Processing

The calibration curves of ELISA or ICA were plotted as dependencies of OD (ELISA) or the color intensity of the analytical zone (ICA) versus the analyte concentrations and fitted using Origin 7.5 software (OriginLab, Northampton, MA, USA) software. All measurements were made in triplicate. The limit of detection (LOD) of the ICA was interpreted as the minimum porcine TnI and RACI concentrations causing a reliable colored analytical zone.

## 3. Results and Discussion

### 3.1. Obtaining and Characterization of Reagents for ICA

**Figure 1:**
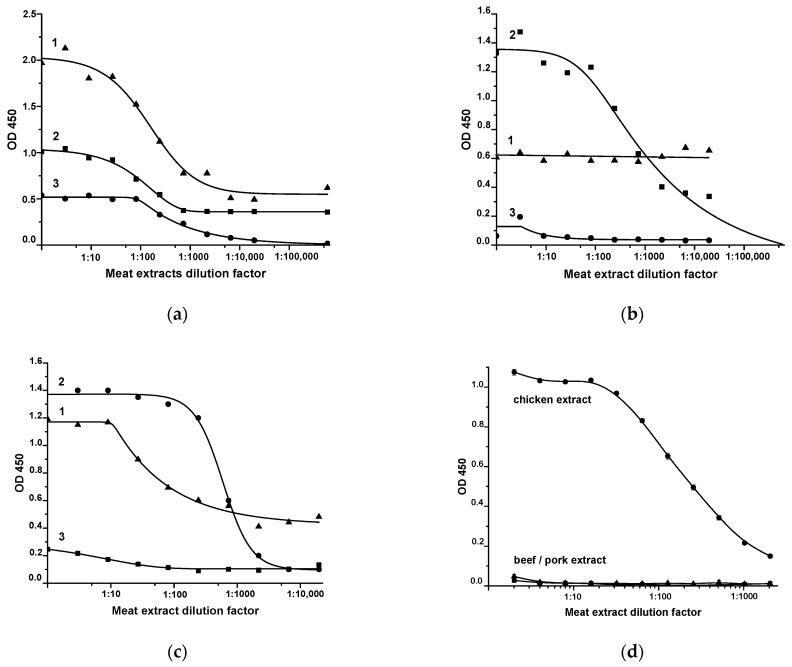
Sandwich ELISA of TnI (**a**–**c**) and IgY (**d**) using different combinations of immunoreactants. On *x* axis—dilution of tested meat extracts, on *y* axis—registered optical density at 450 nm in microplate wells (*n* = 3) reflecting formation of sandwich immune complexes [immobilized antibody—antigen—biotinylated antibody—streptavidin-peroxidase]. (**a**)—immobilized MAb 7G2, (**b**)—immobilized MAb 6F9, (**c**)—immobilized MAb C5. 1—MAb C5-biotin; 2—Mab 7G2-biotin; 3—Mab 6F9-biotin.

The immunoreagents were first characterized via sandwich ELISA. This immunoassay format is suitable for the evaluation of native antigens with at least two epitopes [31]. The applicability of the MAb clones (7G2, 6F9, C5), i.e., acceptable orientation of their binding sites, was tested using porcine skeletal TnI. As can be seen from Figure 1, when MAb 7G2 is immobilized in microplate wells, TnI can be detected in the pork extract using 7G2-biotin, 6F9-biotin, and C5-biotin (Figure 1a). When immobilizing MAb 6F9, detection of TnI is possible only with 7G2-biotin (Figure 1b), and upon immobilization of MAb C5—with 7G2-biotin and C5-biotin (Figure 1c). All of the listed combinations did not bind TnI of birds (chicken, turkey). The use of RACI immobilized in the microplate wells and the RACI-biotin conjugate in the sandwich ELISA made it possible to specifically detect chicken IgY [24]. No interaction with extracts of mammal meat (pork, beef) was observed (Figure 1d).

**Figure 2:**
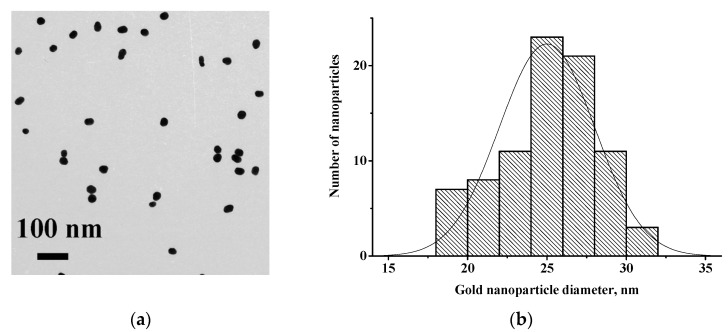
Microphotograph of GNPs obtained by TEM with the indicated scale (**a**); histogram of distribution of GNPs’ diameters (**b**).

The obtained gold nanoparticles (GNPs) had an average size (*n* = 84) of 25.0 ± 3.0 nm (range of variation from 18.4 nm to 30.5 nm) with a degree of ellipticity of 1.21 ± 0.13 (Figure 2). According to microscopic data, the GNPs in the resulting colloidal solution did not aggregate. The solution was also stable when stored at 4 °C; no color change or precipitation was observed for at least two months. For conjugation with the GNPs, antibody concentrations were chosen, which were used in the earlier immunoassay developments [24,26]. Namely, concentrations of monoclonal antibodies 7G2, 6F9, and C5 were 5 μg/mL, and concentration of polyclonal antibodies RACI was 10 μg/mL. Moreover, mixed 7G2 + RACI, 6F9 + RACI, and C5 + RACI preparations with different ratios of the antibodies were conjugated with GNPs.

### 3.2. Development of Individual ICAs

First, test systems for individual analytes were considered. The color intensity of the analytical zone is considered as the assay result reflecting the content of target compound(s) in the tested sample and is named below as the ICA response.

**Figure 3:**
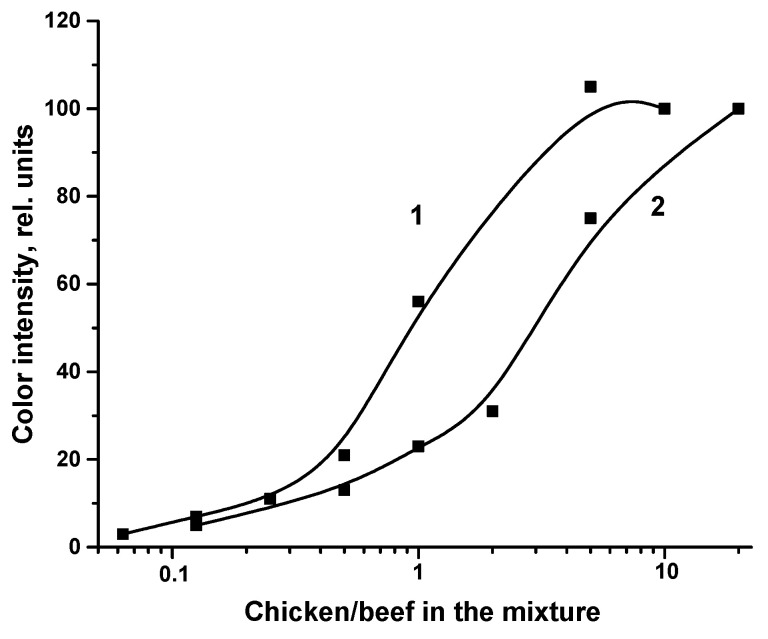
Dependence of the ICA response on the amount (%) of chicken (1) or beef (2) in the meat mixture (*n* = 3).

For the detection of skeletal TnI, MAb 6F9 (2.5 mg/mL) was immobilized in the analytical zone, and MAb 7G2 was conjugated with GNPs [26]. Extracts of meat mixtures were tested, in which 20%, 5%, 2%, 1%, and 0.5% minced beef were added to the minced chicken. As can be seen from Figure 3, significant ICA responses appeared when testing minced chicken extracts containing 1% beef or more.

For the detection of IgY as a specific compound of chicken meat, RACI immobilized in the analytical zone at a concentration of 1 mg/mL and RACI conjugated with GNPs were used for the detection [24]. Extracts of meat mixtures were tested, in which 10%, 5%, 1%, 0.5%, 0.25%, 0.125%, and 0.063% chicken mince were added to minced pork. Significant ICA responses appeared when testing extracts containing 0.25% or more minced chicken meat.

Two curves presented in Figure 3 indicate that when analyzing meat mixtures with the same percentage of target meat (beef or chicken), the ICA response for IgY detection is significantly higher than for TnI detection.

### 3.3. Development of Combined ICA

The proposed combined system for the control of total meat content is based on the simultaneous application of monoclonal antibodies specific to skeletal TnI, which allow the detection of mammalian meat, and rabbit anti-chicken polyclonal immunoglobulins G (RACI), which specifically detect poultry meat. The assay involves the use of a mixture of antibodies specific to TnI and IgY, immobilized in the analytical zone and conjugated with GNPs.

**Figure 4:**
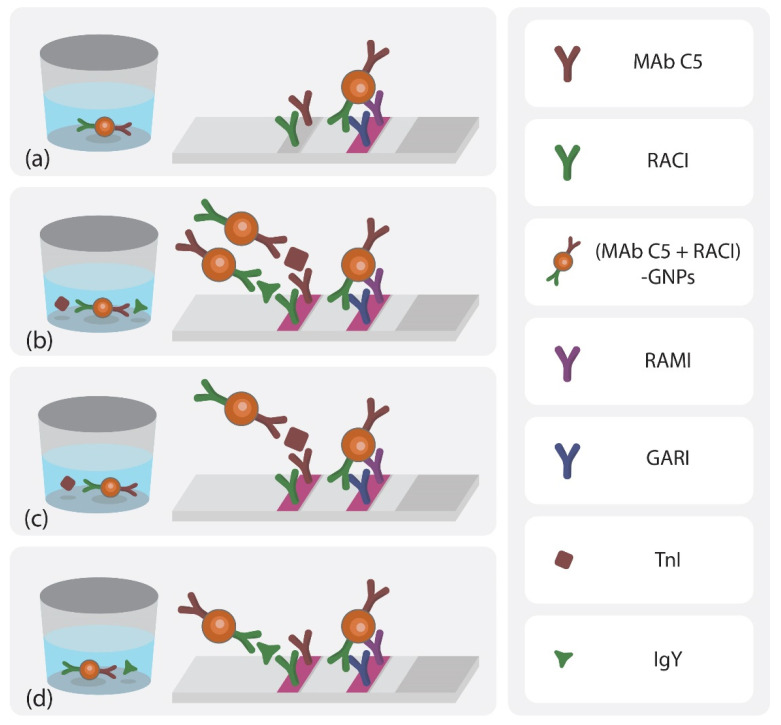
Scheme of the realized sandwich ICA for total meat control. Monoclonal antibodies against mammalian troponin I (MAb C5) and rabbit anti-chicken immunoglobulins G (RACI) were immobilized in the analytical zone of the test strip. In the control zone—rabbit anti-mouse immunoglobulins (RAMI) and goat anti-rabbit immunoglobulins (GARI). The conjugate of MAb C5 and RACI with gold nanoparticles ((C5 + RACI)-GNPs) was mixed with the sample, incubated for 3 min and the test strip was immersed in the mixture. The following combinations of the detected analytes in the sample are demonstrated: (**a**)—absence of TnI (mammalian meat biomarker) and IgY (poultry meat biomarker); (**b**)—presence of TnI and IgY; (**c**)—presence of only TnI; (**d**)—presence of only IgY.

The testing of various combinations of antibodies shows that MAb 7G2 binds to RACI. As a result, the color develops in the analytical zone in the absence of antigen. This excludes the use of MAb 7G2 in the analytical zone or in a conjugate with GNPs. The use of MAb 6F9 on the membrane or in a conjugate with GNPs makes it possible to detect TnI, but these antibodies do not interact with pork extracts, which is consistent with the data obtained by ELISA (Figure 1c). Thus, the only option from the combinations of anti-TnI antibodies for determining total meat is the use of MAb C5 in the analytical zone and in the conjugate with GNPs (Figure 4).

The (C5 + RACI)-GNPs conjugate was mixed with the sample in a microplate well and incubated for 3 min. Then the test strip with MAb C5 and RACI in the analytical zone was immersed in the mixture. If both TnI and IgY were absent in the sample, then the analytical zone was not stained (Figure 4a). If at least one antigen was present, then specific antibodies MAb C5 and RACI bound the corresponding antigen, and then the (MAb C5 + RACI)-GNPs conjugate was included in the formed immune complex, which led to the ICA response (Figure 4b–d).

**Figure 5:**
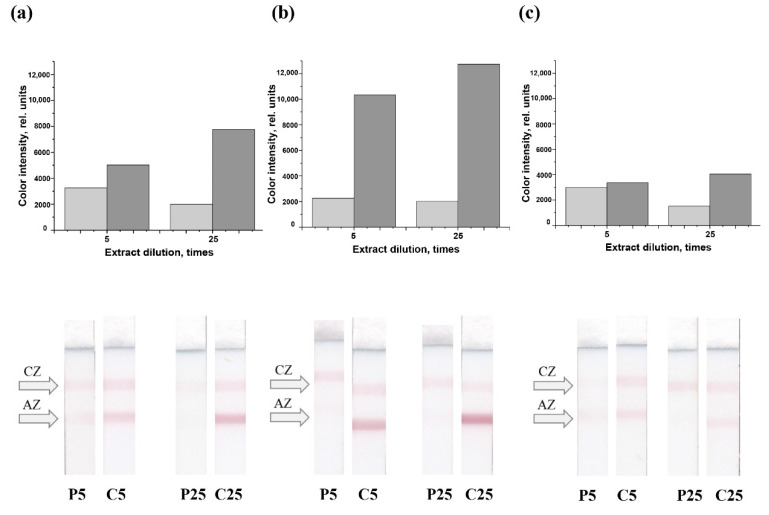
ICA responses (*n* = 3) for 5-fold and 25-fold diluted extracts of pork (gray columns, P5 and P25 test strips) and chicken meat (dark gray columns, C5 and C25 test strips) for (C5 + RACI)-GNPs conjugates of different compositions in which the weight/weight ratio of MAb C5 and RACI in the mixed solution for immobilization was 50/50% (**a**), 20/80% (**b**), and 80/20% (**c**). AZ—analytical zone, CZ—control zone.

When applying a mixture of C5 antibodies (1 mg/mL) with RACI (1 mg/mL) in the analytical zone and using conjugates of different compositions, dependences of the ICA response on the dilution of pork and chicken meat extracts were obtained. As can be seen from Figure 5, the response depends on the number of specific antibodies in the conjugate. At the same time, the response is significantly higher in the analysis of chicken meat extract compared to the same dilutions of pork extract.

It was noted that the application of complete test strips with the lower membrane (for sample absorption) caused variable conditions of solution soaking, and the ICA response may vary significantly. Removal of this membrane excluded these problems and also increased the ICA response, which made it possible to reduce the amount of (C5 + RACI)-GNPs conjugate used per analysis from 2 to 0.5 µL. Therefore, further assays were carried out using shortened tests. In addition, the time of test strip incubation in the reaction mixture was decreased from 20 to 15 min.

### 3.4. Choosing Assay Parameters for Total Meat Content Estimation

Optimization of the C5 + RACI antibodies concentrations of in the analytical zone and the composition of the conjugates of C5 + RACI antibodies with GNPs was carried out to maximally converge the calibration curves obtained for testing individual extracts from pork and chicken meat. The ICA response in the analysis of meat mixtures should have close values and not depend on the ratio of pork and chicken meat.

The best results were obtained using a shortened test system, in the analytical zone of which MAb C5 (1 mg/mL) and RACI (1 mg/mL) were immobilized, and a conjugate (MAb C5 + RACI)-GNPs (80/20%). Characterization of extracts of model meat mixtures, in which the ratios of minced chicken and pork were 25/75, 50/50, 75/25, 90/10, and 95/5, showed high reproducibility of the ICA response for extracts of different compositions when diluted 100 times (Table 1). The results obtained allow recommendation of this test system for total meat analysis.

### 3.5. Analysis of Meat Extracts of Different Compositions

**Figure 6:**
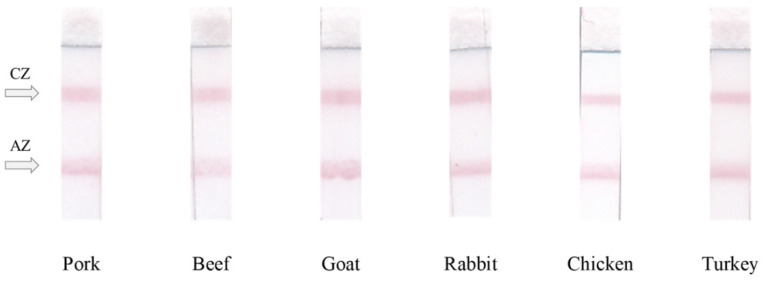
The appearance of the test strips when testing meat extracts from different species. AZ—analytical zone, CZ—control zone.

The possibility of efficient detection of the same ICA response for meat from different sources by the developed combined test was tested. Figure 6 demonstrates the obtained close responses for mammalian (pork, beef, goat, rabbit) and poultry (chicken, turkey) meat.

After this, the developed test systems were used to characterize the extracts of meat mixtures, in which the ratio of minced chicken and pork, minced chicken and beef, and minced chicken and rabbit was 25/75, 50/50, 75/25, 90/10, and 95/5. As can be seen from the results presented in Table 2, the ICA responses for meat mixtures had similar values and do not depend on the ratio of poultry (chicken) meat and mammalian (pork, beef, rabbit) meat in the analyzed samples. The approbation of developed tests for meat/non-meat mixtures demonstrated that the minimal detectable content of meat in mixed samples was 0.1%. High reproducibility of the ICA responses for extracts of different compositions at a dilution of 100 times was shown. RSD of the ICA responses was not more than 10.1%.

Although the presented study is limited by consideration of raw meat samples, the field of application of the proposed concept may be extended. Moreover, our previous developments of ICA using the same immunoreactants against TnI [26] and IgY [24] demonstrated that the recognized antigenic structures are stored in final processed meat products. However, the use of the developed test system to control total content of meat sources of different origins in processed meat products needs additional study.

## 4. Conclusions

A method for immunochromatographic determination of total meat content has been developed. The test system is based on the simultaneous use of antibodies, which specifically interact with mammalian skeletal troponin I (beef, pork, rabbit, lamb), and antibodies, which specifically detect poultry meat (chicken, turkey). The use of a mixture of the antibodies in the analytical zone of the test strip and for conjugation with gold nanoparticles makes it possible to determine the total meat in mixtures of different sources. The chosen reactants provided high reproducibility of the color intensity of the analytical zone for extracts of different compositions but with the same total content of meat sources. The given reasons allow recommending this test system as a quick and simple in use tool for finding deviations of meat-containing products from their declared recipes. The time needed to complete the full cycle test of meat sample(s) is 50 min; it includes 35 min of sample preparation and 15 min of immunochromatographic detection. To make decisions in the further practical application of the test system, it is possible either to compare the ICA response for the sample being characterized and the standard preparation visually, or to quantitatively register the ICA response using existing portable photometric detectors, including smartphones.

## Figures and Tables

**Table 1:** ICA responses in the analysis of mixtures of chicken and pork minces.

Chicken Meat/Porcine Meat Ratio, %	ICA Responses (*n* = 3)
25/75	3181 ± 183
50/50	2961 ± 156
75/25	3172 ± 64
90/10	3144 ± 177
95/5	3112 ± 182

**Table 2:** ICA responses for meat mixtures of different compositions.

Chicken Meat/Mammalian Meat Ratio, %	ICA Response (*n* = 3)		
	Chicken/Porcine	Chicken/Beef	Chicken/Rabbit
25/75	4677 ± 309	4205 ± 239	4261 ± 243
50/50	4673 ± 271	4342 ± 129	4252 ± 225
75/25	4621 ± 259	4348 ± 289	4760 ± 309
90/10	4145 ± 235	4332 ± 115	4515 ± 452
95/5	4484 ± 430	4317 ± 208	4298 ± 222

## Data Availability

The data presented in this study are available on request from the corresponding author.

## References

[1] Pereira P.M., Vicente A.F. (2013). Meat nutritional composition and nutritive role in the human diet. Meat Sci..

[2] Xie Y., Ma Y., Cai L., Jiang S., Li C. (2022). Reconsidering meat intake and human health: A review of current research. Mol. Nutr. Food Res..

[3] Kulczyński B., Sidor A., Gramza-Michałowska A. (2019). Characteristics of selected antioxidative and bioactive compounds in meat and animal origin products. Antioxidants.

[4] Stangierski J., Lesnierowski G. (2015). Nutritional and health-promoting aspects of poultry meat and its processed products. World’s Poult. Sci. J..

[5] Yousefi M., Khorshidian N., Hosseini H. (2018). An overview of the functionality of inulin in meat and poultry products. Nutr. Food Sci..

[6] Li Y.C., Liu S.Y., Meng F.B., Liu D.Y., Zhang Y., Wang W., Zhang J.M. (2020). Comparative review and the recent progress in detection technologies of meat product adulteration. Compr. Rev. Food Sci. Food Saf..

[7] González N., Marquès M., Nadal M., Domingo J.L. (2020). Meat consumption: Which are the current global risks? A review of recent (2010–2020) evidences. Food Res. Int..

[8] Jurica K., Karaconji I.B., Lasić D., Kovacević D.B., Putnik P. (2021). Unauthorized food manipulation as a criminal offense: Food authenticity, legal frameworks, analytical tools and cases. Foods.

[9] Canan C., Adamante D., Kalschne D., Corso M., Zanatta E.R. (2020). Soy protein: A food allergen frequently used in the preparation of meat products. Rev. Chil. Nutr..

[10] Alikord M., Momtaz H., Keramat J., Kadivar M., Rad A.H. (2018). Species identification and animal authentication in meatproducts: A review. Food Measur..

[11] Geiker N.R.W., Bertram H.C., Mejborn H., Dragsted L.O., Kristensen L., Carrascal J.R., Bügel S., Astrup A. (2021). Meat and human health—Current knowledge and research gaps. Foods.

[12] Haddad M. (2021). The impact of CB1 receptor on nuclear receptors in skeletal muscle cells. Pathophysiology.

[13] Haddad M. (2021). Impact of adenosine A2 receptor ligands on BCL2 expression in skeletal muscle cells. Appl. Sci..

[14] Chernukha I.M., Vostrikova N.L., Khvostov D.V., Zvereva E.A., Taranova N.A., Zherdev A.V. (2019). Methods of identification of muscle tissue in meat products. Prerequisites for creating a multi–level control system. Theory Pract. Meat Proces..

[15] Stachniuk A., Sumara A., Montowska M., Fornal E. (2021). Liquid chromatography–mass spectrometry bottom-up proteomic methods in animal species analysis of processed meat for food authentication and the detection of adulterations. Mass Spectrom. Rev..

[16] Kumar Y., Narsaiah K. (2021). Rapid point-of-care testing methods/devices for meat species identification: A review. Compr. Rev. Food Sci. Food Saf..

[17] Harlina P.W., Maritha V., Musfiroh I., Huda S., Sukri N., Muchtaridi M. (2022). Possibilities of liquid chromatography mass spectrometry (LC-MS)-based metabolomics and lipidomics in the authentication of meat products: A mini review. Food Sci. Anim. Resour..

[18] Dzantiev B.B., Byzova N.A., Urusov A.E., Zherdev A.V. (2014). Immunochromatographic methods in food analysis. Trend Anal. Chem..

[19] Chen F.C., Hsieh Y.P. (2002). Porcine troponin I: A thermostable species marker protein. Meat Sci..

[20] Masiri J., Benoit L., Barrios-Lopez B., Thienes C., Meshgi M., Agapov A., Dobritsa A., Nadala C., Samadpour M. (2016). Development and validation of a rapid test system for detection of pork meat and collagen residues. Meat Sci..

[21] Zvereva E.A., Kovalev L.I., Ivanov A.V., Kovaleva M.A., Zherdev A.V., Shishkin S.S., Lisitsyn A.B., Chernukha I.M., Dzantiev B.B. (2015). Enzyme immunoassay and proteomic characterization of troponin I as a marker of mammalian muscle compounds in raw meat and some meat products. Meat Sci..

[22] Kotoura S., Murakami-Yamaguchi Y., Kizu K., Nakamura M., Fuchu H., Kiyotaka Miake K., Sugiyama M., Narita H. (2012). Establishment of a sandwich ELISA for the determination of beef content in processed foods by using monoclonal antibodies to myoglobin. Food Agric. Immunol..

[23] Jiang X., Rao Q., Mittl K., Hsieh Y.P. (2020). Monoclonal antibody-based sandwich ELISA for the detection of mammalian meats. Food Control.

[24] Hendrickson O.D., Zvereva E.A., Vostrikova N.L., Chernukha I.M., Dzantiev B.B., Zherdev A.V. (2021). Lateral flow immunoassay for sensitive detection of undeclared chicken meat in meat products. Food Chem..

[25] Kuswandi B., Gani A.A., Ahmad M. (2017). Immuno strip test for detection of pork adulteration in cooked meatballs. Food Biosci..

[26] Zvereva E.A., Popravko D.S., Hendrickson O.D., Vostrikova N.L., Chernukha I.M., Dzantiev B.B., Zherdev A.V. (2020). Lateral flow immunoassay to detect the addition of beef, pork, lamb, and horse muscles in raw meat mixtures and finished meat products. Foods.

[27] Hossain M.A.M., Uddin S.M.K., Sultana S., Wahab Y.A., Sagadevan S., Johan M.R., Eaqub A. (2022). Authentication of Halal and Kosher meat and meat products: Analytical approaches, current progresses and future prospects. Crit. Rev. Food Sci. Nutr..

[28] Afzaal M., Saeed F., Hussain M., Shahid F., Siddeeg A., Al-Farga A. (2022). Proteomics as a promising biomarker in food authentication, quality and safety: A review. Food Sci. Nutr..

[29] Bayer E.A., Wilchek M. (1990). Protein biotinylation. Meth. Enzymol..

[30] Frens G. (1973). Controlled nucleation for the regulation of the particle size in monodisperse gold suspensions. Nat. Phys. Sci..

[31] Wild D.G. (2013). Immunoassay Handbook: Theory and Applications of Ligand Binding, ELISA and Related Techniques.

